# In-hospital and one-year outcomes in cancer patients receiving percutaneous coronary intervention for acute myocardial infarction: A real-world study

**DOI:** 10.3389/fcvm.2022.1005473

**Published:** 2023-02-07

**Authors:** Manyun Tang, Yidan Wang, Xiangqi Cao, John D. Day, Hui Liu, Chaofeng Sun, Guoliang Li

**Affiliations:** ^1^Department of Hepatobiliary Surgery, The First Affiliated Hospital of Xi’an Jiaotong University, Xi’an, China; ^2^Department of Cardiovascular Medicine, The First Affiliated Hospital of Xi’an Jiaotong University, Xi’an, China; ^3^Stroke Centre and Department of Neurology, The First Affiliated Hospital of Xi’an Jiaotong University, Xi’an, China; ^4^Department of Cardiology, St. Mark’s Hospital, Salt Lake City, UT, United States; ^5^BioBank, The First Affiliated Hospital of Xi’an Jiaotong University, Xi’an, China

**Keywords:** cardio-oncology, acute myocardial infarction, cancer, percutaneous coronary intervention, prognosis

## Abstract

**Background:**

Cancer and ischemic heart disease are the leading causes of mortality. The optimal management for patients with concomitant acute myocardial infarction (AMI) and cancer remains challenging.

**Objective:**

To evaluate in-hospital and 1-year adverse outcomes in cancer patients receiving percutaneous coronary intervention (PCI) to treat AMI.

**Methods:**

This was a single-center, retrospective cohort study, patients with cancer admitted to The First Affiliated Hospital of Xi’an Jiaotong University for AMI and discharged between January 2015 and June 2020 were analyzed. The outcomes were all-cause mortality at 1-year follow up and incidence of in-hospital adverse events, including arrhythmias, heart failure, major bleeding, stroke, and all-cause death.

**Results:**

A total of 119 patients were included, of these, 68 (57.1%) received PCI (PCI group) and 51 (42.9%) did not (non-PCI group). Patients in the PCI group had a lower incidence of in-hospital arrhythmias (22.1 vs. 39.2%; *p* = 0.042), major bleeding (2.9 vs. 15.7%; *p* = 0.013), and all-cause mortality (1.5 vs. 11.8%; *p* = 0.018) than those in non-PCI group. On 1-year follow-up, the PCI group had a lower all-cause mortality than the non-PCI group (log-rank test = 14.65; *p* < 0.001). Multivariable Cox regression showed that PCI is an independent protective factor (adjusted HR = 0.503 [0.243–0.947], *p* = 0.045) for cancer patients who have concomitant AMI.

**Conclusion:**

Cancer patients receiving PCI for AMI had a lower risk of in-hospital adverse events and mortality as well as 1-year all-cause mortality compared to those who refused PCI. Our study therefore supports the use of PCI to improve prognosis of this selected group of patients.

## Introduction

Cardiovascular disease (CVD) and cancer are two leading causes of mortality worldwide, together accounting for nearly 70% of disease-related mortality in developed countries (1). Over the last two decades, cancer-related deaths have declined due to advances in therapeutic interventions (2). Similarly, the deaths from acute myocardial infarction (AMI) have also declined globally, which is associated with an increase in the use of treatments and prevention strategies indicated by guidelines.

However, the number of people suffering from concomitant cancer and CVD is on the rise (3), and the causal relationship between CVD and cancer can be partially attributed to shared risk factors, such as an aging population, obesity, diabetes and smoking (4, 5), and common mechanisms, including pro-inflammatory and hypercoagulable states (6). What is more, the treatment of the cancer itself may also have indirect cardiac risk in that some chemotherapeutic agents used to treat cancer have cardio-toxic effects (7), which are associated with the acute thrombosis, acceleration of atherosclerotic plaque formation and coronary vasospasm culminating in future acute coronary syndromes (ACS) (8).

Currently, few studies have evaluated the early and late outcomes of patients suffering from concomitant cancer and AMI. In addition, patients with a cancer history are often excluded from clinical trials evaluating new therapies for ACS. This may explain why the clinical guidelines concerning treatment options for patients with concomitant CVD and cancer are scarce. Therefore, we have conducted a real-world study to compare the risk factors, in-hospital and 1-year outcomes in a Chinese cohort of cancer patients receiving percutaneous coronary intervention (PCI) and medical therapy to treat AMI.

## Materials and methods

### Study design

This is a single-center, retrospective cohort study, all anonymized clinical data were collected from the Biobank of the First Affiliated Hospital of Xi’an Jiaotong University from January 2015 to June 2020. Sources of patients’ information include medical records, death certificates, and hospital discharge summaries. This study was approved by the Ethics Committee of the First Affiliated Hospital of Xi’an Jiaotong University (No. XJTU1AF2021LSK116), and informed consents were obtained. All methods were performed in accordance with the relevant guidelines and regulations according to the principles expressed in the World Medical Association Declaration of Helsinki.

### Objectives

Patients with AMI and a clear diagnosis of cancer were consecutively enrolled to avoid selection bias. AMI including ST-segment elevation myocardial infarction (STEMI) and non ST-segment elevation myocardial infarction (NSTEMI), which was defined based on the fourth universal definition of myocardial infarction (9). Cancer patients indicated those who had active cancer or a history of cancer. Types of cancer included breast, gastrointestinal tract, prostate, urologic, liver, respiratory tract, gynecological, and other locations. Cancer and AMI were identified by International Classification of Diseases codes for medical settings and then validated by medical record review.

Exclusion criteria were: (1) age <18 years old; (2) previous myocardial infarction and prior PCI; (3) preexisting serious comorbidities: acquired immunodeficiency syndrome, advanced heart disease, dementia, liver failure, severe renal impairment (estimated glomerular filtration rate <30 ml/min/1⋅73 m^2^); (4) lack of clinical data; and (5) unwillingness to participate in the study.

### Data collection

All data on baseline characteristics, including demographics [age, sex, body mass index (BMI), and Charlson Comorbidity Index (CCI) score], comorbidities (hypertension, diabetes, hyperlipidemia, and smoking), medical history (history of cancer, myocardial infarction, arrhythmias, heart failure, major bleeding, stroke, and death), and laboratory examination were obtained from the electronic medical record system of the hospital and recorded using a standardized protocol. Information of in-hospital treatment, including PCI and medications [anticoagulant drugs, antiplatelet drugs, angiotensin-converting enzyme inhibitors (ACEIs) or angiotensin II receptor blockers (ARBs), beta-blockers, potassium-sparing diuretics, and lipid-lowering drugs], was also collected.

### Follow-up and outcomes

Patients were retrospectively followed. The primary endpoint of this study was defined as all-cause mortality at 1-year follow up. Survival status after discharge was prioritized to obtain through the follow up in the outpatient department. For patients unable to follow up at the hospital, we obtained their survival information through telephone contact with patients themselves or their first-degree relatives. The secondary endpoint was defined as the incidence of in-hospital major adverse events, including arrhythmias, heart failure, stroke, all-cause death, and major bleeding. Arrhythmias, including atrial fibrillation, premature atrial contractions, ventricular fibrillation, premature ventricular contractions, ventricular tachycardia, and high-grade atrioventricular block. Major bleeding was defined as fatal bleeding, and/or symptomatic bleeding in a critical area or organ, such as intracranial, intraspinal, intraocular, retroperitoneal, intra-articular or pericardial, or intramuscular with compartment syndrome, and/or bleeding causing a fall in hemoglobin level of 20 g/l (1.24 mmol/l) or more, or leading to transfusion of two or more units of whole blood or red cells (10). All-cause death was defined as death from any cause during hospitalization.

### Statistical analysis

All statistical analysis was performed using the IBM SPSS Statistics version 26.0. Categorical variables were described as frequencies and percentages and compared using Chi-squared test. Continuous variables were described as the mean (x̄) and standard deviation (SD) and compared using independent *t*-tests or non-parametric Mann–Whitney tests. Kolmogorov–Smirnov test was used to assess the distribution of continuous variables. Whether PCI was an independent correlate of incidence of in-hospital adverse events was determined using Binary logistic regression analysis. Survival curves were generated using the Kaplan–Meier method, and were compared by the use of log-rank tests. Multivariate analysis for 1-year mortality was carried out using Cox proportional hazards modeling, using demographic characteristics, risk factors, and previous medical history as covariables. Two-sided *p* < 0.05 was considered statistically significant.

## Results

### Baseline characteristics

**FIGURE 1:**
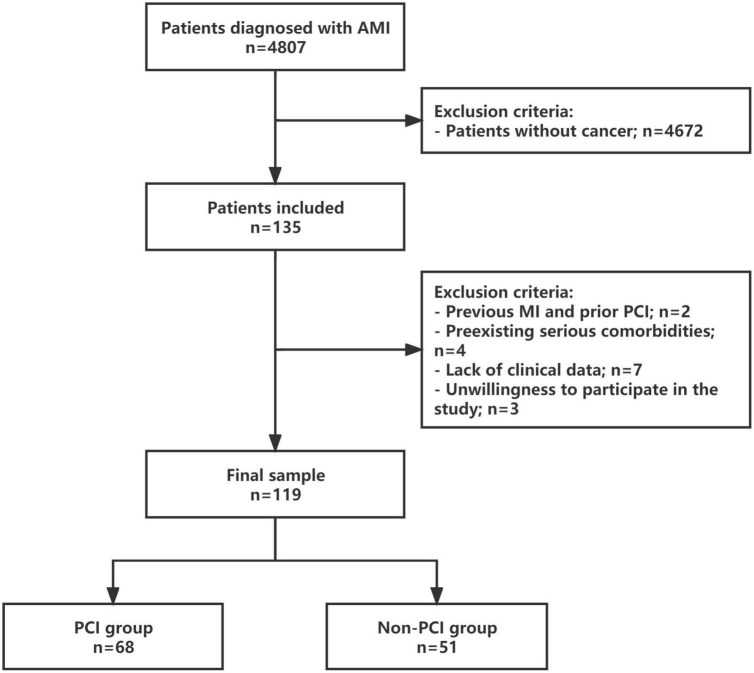
Study protocol. Flowchart of the selection process and dropouts of the current study. AMI, acute myocardial infarction; MI, myocardial infarction; PCI, percutaneous coronary intervention.

Of the 4,807 AMI patients initially recruited to the study, 135 had cancer. Through a detailed screening, 119 consecutive patients were finally enrolled to this study, and divided into the PCI group and non-PCI group (Figure 1).

**FIGURE 2:**
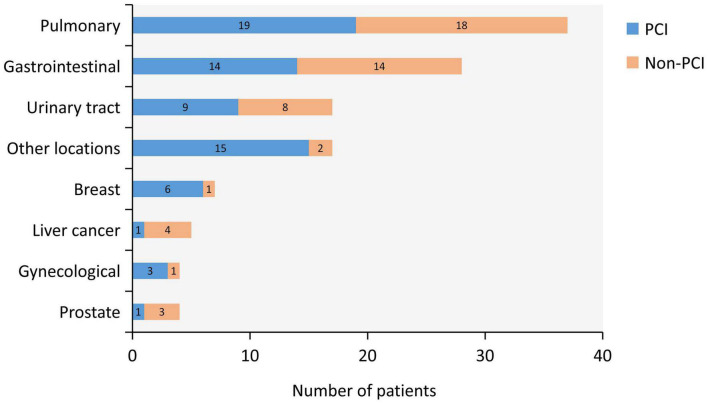
Type of Cancer. Type of cancer and distribution between percutaneous coronary intervention (PCI) group (blue) and non-PCI group (orange) are shown. Most common cancer types were pulmonary, gastrointestinal, urinary tract, and other locations.

Among these 119 patients, cancer was found in a wide range of locations and were as follows: breast cancer in seven patients (5.8%), gastrointestinal cancer in 28 (23.5%), prostate cancer in 4 (3.4%), urologic neoplasms in 17 (14.3%), liver cancer in 5 (4.2%), pulmonary cancer in 37 (31.1%), gynecological cancer in 4 (3.4%), and other locations in 17 (14.3%). Most common cancer types were pulmonary, gastrointestinal, urinary tract, and other locations (Figure 2).

According to the clinical characteristics (Table 1), patients undergoing PCI had a higher mean BMI (23.26 vs. 22.11, *p* < 0.001) and lower mean CCI score (5.13 vs. 7.92, *p* < 0.001), age, sex and comorbidities, including hypertension, diabetes, dyslipidemia, and smoking, were similar between the two groups (all *p* > 0.05) (Table 1). No significant differences were observed in types of AMI, frequency of Killip class ≥2, serum troponin T, creatine kinase, and creatine kinase-MB levels (all *p* > 0.05). Recent cancer diagnosis within 1-year (64.7 vs. 23.5%, *p* < 0.001) or cancer with metastasis (29.4 vs. 10.3%, *p* = 0.008) were more common in the non-PCI group. More patients received chemotherapy in the non-PCI group than in the PCI group (29.4 vs. 11.8%, *p* = 0.016), with comparable rates of those receiving radiotherapy.

**TABLE 1:** Baseline characteristics.

	Overall *N* = 119	PCI group *N* = 68	Non-PCI group *N* = 51	*P*-value
Age (years)	66.42 ± 11.05	65.57 ± 11.39	67.55 ± 10.58	0.580
Male [n (%)]	92 (77.3)	52 (76.5)	40 (78.4)	0.800
BMI (kg/m^2^)	22.77 ± 2.72	23.26 ± 2.48	22.11 ± 2.90	<0.001
CCI	6.33 ± 2.66	5.13 ± 1.70	7.92 ± 2.87	<0.001
**Comorbidities**				
Hypertension [n (%)]	67 (55.4)	38 (54.5)	29 (56.9)	0.778
Diabetes mellitus [n (%)]	31 (26.1)	16 (23.5)	15 (29.4)	0.469
Dyslipidemia [n (%)]	6 (5.0)	2 (2.9)	4 (7.8)	0.227
Tobacco use [n (%)]	37 (31.1)	23 (33.8)	14 (27.5)	0.457
**Cancer**				
Diagnosis within 1 Year [n (%)]	49 (41.2)	16 (23.5)	33 (64.7)	<0.001
Metastasis [n (%)]	22 (18.5)	7 (10.3)	15 (29.4)	0.008
Chemotherapy [n (%)]	20 (16.8)	6 (8.8)	14 (27.5)	0.007
Radiotherapy [n (%)]	9 (7.6)	5 (7.4)	4 (7.8)	1.000
**Acute myocardial infarction**				
STEMI [n (%)]	30 (25.2)	19 (27.9)	11 (21.6)	0.428
NSTEMI [n (%)]	89 (74.8)	49 (72.1)	40 (78.4)	0.428
Killip class ≥2 [n (%)]	39 (32.8)	20 (29.4)	19 (37.3)	0.367
Troponin T (ng/mL)	1.22 ± 1.81	1.33 ± 2.00	1.08 ± 1.53	0.250
Creatine kinase (μ/L)	479.48 ± 808.88	589.62 ± 987.60	332.62 ± 445.70	0.245
Creatine kinase-MB (μ/L)	56.01 ± 88.86	70.43 ± 111.74	36.77 ± 35.25	0.249
**Laboratory data**				
Hemoglobin (g/L)	126.30 ± 23.76	132.93 ± 19.70	117.47 ± 25.94	0.001
Platelet count (10^9^/L)	211.40 ± 77.06	214.54 ± 69.09	207.20 ± 87.10	0.293
White blood cell (10^9^/L)	8.84 ± 4.00	9.06 ± 3.57	8.56 ± 4.45	0.172
CRP (mg/L)	23.77 ± 28.56	14.21 ± 19.82	32.75 ± 34.06	<0.001
BNP (pg/mL)	4461.20 ± 7959.58	3319.01 ± 6630.63	5984.11 ± 9298.61	0.016
LDL (mmol/L)	2.27 ± 0.74	2.25 ± 0.86	2.30 ± 0.56	0.213
Triglyceride (mmol/L)	1.54 ± 1.02	1.57 ± 1.23	1.49 ± 0.56	0.074
Total cholesterol (mmol/L)	3.94 ± 1.10	4.00 ± 1.16	3.85 ± 1.03	0.757
D-dimer (mg/L)	3.69 ± 9.04	2.03 ± 6.73	5.91 ± 11.12	<0.001
PT (s)	14.09 ± 1.50	13.82 ± 1.34	14.43 ± 1.63	0.009
APTT (s)	36.95 ± 4.39	36.25 ± 4.36	37.89 ± 4.29	0.003
eGFR	78.60 ± 19.84	85.53 ± 18.79	69.35 ± 17.43	<0.001
Creatinine (μmol/L)	97.98 ± 112.61	85.41 ± 102.91	114.73 ± 123.43	0.075
ASL (μ/L)	74.06 ± 111.47	87.96 ± 138.50	55.52 ± 54.84	0.082
ALT (μ/L)	46.36 ± 163.02	59.35 ± 213.89	29.03 ± 29.68	0.317
AST/ALT	2.40 ± 3.01	2.36 ± 2.38	2.46 ± 3.72	0.860
Albumin (g/L)	37.26 ± 5.50	38.08 ± 4.09	36.17 ± 6.84	0.081
LVEF (%)	53.03 ± 10.28	52.56 ± 9.04	53.80 ± 11.44	0.509
**Medications**				
Anticoagulant drugs [n (%)]	34 (28.6)	19 (27.9)	15 (29.4)	0.861
DAPT [n (%)]	84 (70.6)	62 (91.2)	26 (51.0)	<0.001
Aspirin [n (%)]	84 (70.6)	58 (85.3)	26 (51.0)	<0.001
Clopidogrel [n (%)]	72 (60.5)	45 (66.2)	27 (52.9)	0.144
Ticagrelor [n (%)]	29 (24.4)	25 (36.8)	4 (7.8)	<0.001
Tirofiban [n (%)]	38 (31.9)	31 (45.6)	7 (13.7)	<0.001
ACEIs/ARBs [n (%)]	65 (54.6)	45 (66.2)	20 (39.2)	0.003
β-blockers [n (%)]	78 (65.5)	54 (79.4)	24 (20.6)	<0.001
Potassium-sparing diuretics [n (%)]	38 (31.9)	24 (35.3)	14 (27.5)	0.364
Lipid lowering drugs [n (%)]	87 (73.1)	59 (86.8)	28 (54.9)	<0.001

Values are presented as mean ± SD or n (%).

PCI, percutaneous coronary intervention; BMI, body mass index; CCI, charlson comorbidity index; STEMI, ST-segment elevation myocardial infarction; NSTEMI, non-ST-segment elevation myocardial infarction; BNP, brain natriuretic peptide; CRP, C-reactive protein; LDL, low density lipoprotein; PT, prothrombin time; APTT, activated partial thromboplastin time; eGFR, estimated glomerular filtration rate; AST, aspartate aminotransferase; ALT, alanine transaminase; LVEF, left ventricular ejection fraction; DAPT, dual antiplatelet therapy; ACEIs, angiotensin-Converting Enzyme Inhibitors; ARBs, angiotensin receptor blockers.

The patients undergoing PCI had higher serum levels of hemoglobin and lower serum levels of brain natriuretic peptide (BNP), C- reactive protein (CRP) and D-dimer than those who did not undergo PCI (all *p* < 0.01). The estimated glomerular filtration rate (eGFR) was lower and prothrombin time (PT) as well as activated partial thromboplastin time (APTT) were longer in the non-PCI group when compared with the PCI group. However, no significant difference was found in the liver function and lipid tests between the two groups, including aspartate aminotransferase (AST), alanine aminotransferase (ALT), AST/ALT, albumin, low density lipoprotein, triglyceride, cholesterol, and lactate dehydrogenase (LDH) (Table 1).

Regarding medications, dual antiplatelet therapy (DAPT) was more commonly observed in patients who underwent PCI (*p* < 0.001) and there was a significant difference in the antiplatelet agents used, including the use of aspirin, ticagrelor and tirofiban, with higher medication use in the PCI group (all *p* < 0.001). In addition, the number of patients using ACEIs/ARBs, beta blockers, and lipid lowering drugs in the PCI group was also significantly higher than that in the non-PCI group (all *p* < 0.01). However, the use of anticoagulant drugs, clopidogrel, and potassium-sparing diuretics were similar between two groups (Table 1).

### In-hospital outcomes

Compared with patients in the non-PCI group, patients in the PCI group had a significantly lower incidence of arrhythmias (22.1 vs. 39.2%, *p* = 0.042; OR = 0.439 [0.197–0.979]), major bleeding (2.9 vs. 15.7%, *p* = 0.013; OR = 0.163 [0.033–0.804]), and all-cause death (1.5 vs. 11.8%, *p* = 0.018; OR = 0.112 [0.013–0.916]). However, the incidence of heart failure and stroke was not significantly different between the two groups (Table 2).

**TABLE 2:** In-hospital outcomes among two groups.

	Overall *N* = 119	PCI *N* = 68	Non-PCI *N* = 51	Crude OR	95% CI	*P*-value
Arrhythmia [n (%)]	35 (29.4)	15 (22.1)	20 (39.2)	0.439	0.197–0.979	0.042
Heart failure [n (%)]	19 (16)	8 (11.8)	11 (21.6)	0.485	0.179–1.311	0.148
Major bleeding [n (%)]	10 (8.4)	2 (2.9)	8 (15.7)	0.163	0.033–0.804	0.013
Stroke [n (%)]	19 (16)	11 (16.2)	8 (15.7)	1.037	0.384–2.800	0.942
Death [n (%)]	7 (5.9)	1 (1.5)	6 (11.8)	0.112	0.013–0.916	0.018

Values are presented as n (%).

PCI, percutaneous coronary intervention; OR, odds ratios; CI, confidence interval.

### Outcomes at 1-year follow-up

**FIGURE 3:**
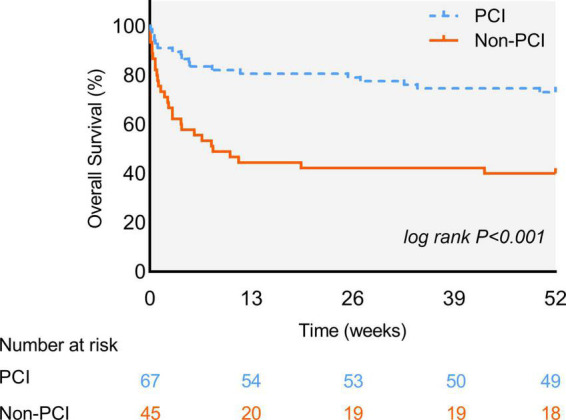
The 1-Year Kaplan-Meier survival curves. Survival rate between patients in percutaneous coronary intervention (PCI) group (blue line) and non-PCI group (orange line). The difference is statistically significant (Log-rank test = 14.65, *p* < 0.001).

During 1-year follow-up, 45 (41.7%) patients died, with lower rate in the PCI group compared to the non-PCI group [18 (27.3%) vs. 27 (64.3%); Log-rank test = 14.65, *p* < 0.001]; (Figure 3). Univariate Cox regression showed that PCI (crude hazard ratio (HR) = 0.326 [0.181–0.589], *p* < 0.001), BMI (crude HR = 0.882 [0.799–0.973], *p* = 0.012), hemoglobin (crude HR = 0.979 [0.967–0.991], *p* = 0.001), eGFR (crude HR = 0.986 [0.974–0.998], *p* = 0.021), and DAPT (crude HR = 0.466 [0.259–0.840], *p* = 0.011) were associated with significantly lower mortality; while CCI score (crude HR = 1.138 [1.027–1.263], *p* = 0.014), cancer diagnosis within 1-year (crude HR = 2.134 [1.193–3.817], *p* = 0.011), chemotherapy (crude HR = 2.462 [1.292–4.693], *p* = 0.006), CRP level (crude HR = 1.01 [1.002–1.018], *p* = 0.015), and D-dimer level (crude HR = 1.041 [1.010–1.074], *p* = 0.010) were associated with significantly higher mortality. However, after adjusting for confounding factors, apart from PCI (adjusted HR = 0.503 [0.243–0.947], *p* = 0.045), the above variables were no longer significant (Table 3), and PCI was identified as a protective factor for patients with concomitant cancer and AMI.

**TABLE 3:** Univariate and multivariate Cox regression analyses for all-cause mortality.

	Univariate analysis			Multivariate analysis		
	Crude HR	95% CI	*P*-value	Adjusted HR*	95% CI	*P*-value
PCI	0.326	0.181–0.589	<0.001	0.503	0.243–0.947	0.045
Male	1.323	0.639–2.742	0.451			
Age	1.012	0.983–1.042	0.430			
BMI	0.882	0.799–0.973	0.012			
CCI	1.138	1.027–1.263	0.014	1.014	0.893–1.151	0.832
Cancer diagnosis within 1 year	2.134	1.193–3.817	0.011	1.524	0.788–2.946	0.210
Metastasis	1.293	0.624–2.680	0.490			
Chemotherapy	2.462	1.292–4.693	0.006	1.781	0.887–3.579	0.105
Hemoglobin	0.979	0.967–0.991	0.001			
White blood cell	1.034	0.962–1.111	0.369			
Creatinine	1.001	0.999–1.003	1.001			
eGFR	0.986	0.974–0.998	0.021			
BNP	1.000	1.000–1.000	0.109			
CRP	1.010	1.002–1.018	0.015			
D-dimer	1.041	1.010–1.074	0.010			
PT	1.135	0.960–1.341	0.137			
APTT	1.051	0.988–1.119	0.115			
DAPT	0.466	0.259–0.840	0.011	0.720	0.376–1.380	0.323

*Multivariable model adjusts for PCI, male, age, BMI, CCI, cancer diagnosis within 1 year, metastasis, chemotherapy, hemoglobin, white blood cell, creatinine, eGFR, BNP, CRP, D-dimer, PT, APTT and DAPT.

HR, hazard ratio; CI, confidence interval; PCI, percutaneous coronary intervention; BMI, body mass index; CCI, charlson comorbidity index; eGFR, estimated glomerular filtration rate; BNP, brain natriuretic peptide; CRP, C-reactive protein; PT, prothrombin time; APTT, activated partial thromboplastin time; DAPT, dual antiplatelet therapy.

## Discussion

The main finding of this study is that cancer patients who received PCI to treat AMI had a better 1-year survival rate than those who did not, suggesting that PCI may be beneficial for treating AMI in cancer patients. In addition, a significant increase in the incidence of arrhythmias, major bleeding and death was observed in patients in the non-PCI group during hospitalization. However, the use of chemotherapy was an independent risk factor affecting the prognosis in our patient cohort. The cross-research focus of cancer and CVD has only recently received more extensive attention, and many areas lacking evidence need to be explored in future research. To the best of our knowledge, this is the first real-world study based on the Chinese population to evaluate in-hospital and 1-year outcomes and risk factors in cancer patients receiving PCI to treat AMI.

As advances in therapeutic interventions for CVD and cancer have improved survival rates (11), it is of great importance to determine the clinical impact of invasive interventions. PCI has emerged as one of the fundamental strategies in the management of acute ischemic syndromes. However, most of the cardiovascular clinical trials systematically excluded cancer patients, and there is limited data from observational studies regarding the prognosis of AMI patients with a history of cancer after PCI. The Dutch multicenter registry study has found that patients with STEMI with a history of cancer have higher cardiac and all-cause 1-year mortality rates than patients without such a history (12). Another study also found that all-cause mortality rates were higher in patients with cancer after PCI in general (13). However, a single-center study reported that there was no survival advantage in patients with STEMI who were treated with PCI compared with the patients who did not have a previous cancer diagnosis (14). These previous studies focus on the effect of history of cancer on the prognosis of patients with AMI. There are few considerations on the interventional treatment strategies of cancer patients with AMI. Our study provides evidence to evaluate the effect of PCI on the early and late outcomes in the setting of AMI, thereby contributing to the field of PCI outcomes where there is limited experience. Our findings demonstrate that patients with concomitant cancer and AMI without receiving PCI have a higher incidence of in-hospital arrhythmias, major bleeding, and mortality. All-cause mortality at 1-year follow-up was also significantly higher in cancer patients who did not undergo PCI to treat AMI.

Management of AMI in cancer patients is quite challenging, guidelines on cardio-oncology suggest that an individualized guideline-based management is urgently needed, which takes cancer status, prognosis, and the patient’s preferences regarding invasive management into account (3). In our experience, individuals with cancer presenting with AMI are relatively hesitant to undergo coronary angiography and PCI. Velders et al. (12) found that patients with history of cancer were less likely to receive PCI. Similarly, our observations were consistent with the previous study, in that only 57.1% of cancer patients were treated with reperfusion therapy whereas 42.9% did not. We also found that the willingness of patients to receive PCI was not related to their age or gender, but to CCI and BMI, which reflect the health status of patients to some extent and influenced their final decision-making process about whether or not to perform an invasive strategy. What is more, patients with a recent cancer diagnosis within 1 year of their AMI or with metastatic cancer were also less likely to undergo a PCI. According to 2017 European Society of Cardiology (ESC) Guidelines for the management of ACS, primary PCI is the preferred reperfusion strategy in patients with STEMI, and fibrinolysis could also be accepted in some circumstances where primary PCI could not be performed immediately (15). However, for patients with NSTEMI, only the coronary stent implantation with DAPT is recommended (16). In this study, there were more NSTEMI in either PCI group or non-PCI group, which, to some extent, explained the higher mortality in patients in non-PCI group.

In a previous study conducted by Ederhy et al. (17), the management of patients with a history of cancer did not differ from those patients free from cancer. In our study, however, significant differences in medications have been highlighted, DAPT was more common in patients undergoing PCI, in line with the more frequent performance of PCI in these patients. The trend toward more prescriptions of ACEIs/ARB, β-blockers and lipid lowering drugs were also observed in patients who underwent PCI. There were no significant differences concerning anticoagulant drugs between the PCI group and the non-PCI group which suggests a different clinical management strategy between cancer patients who underwent PCI and those who did not. As for further screening factors involved in clinical decision-making, hemoglobin, eGFR, BNP at admission and the proportion of cancer diagnosis within 1-year or metastatic cancer may, partially, explain these results.

Chemotherapy was shown to be an independent risk factor for 1-year survival on multivariable Cox regression in our study. Indeed, chemotherapy-induced cardiotoxicity has been well recognized from many studies to be a significant cause of left ventricular dysfunction, heart failure, hypertension, rhythm disturbances, vascular thrombosis, and ischemia, all resulting in a poor prognosis (18). During the follow-up period, the vast majority of patient deaths that occurred within 6 months of discharge had cancer in the year preceding the AMI event and this can be partly explained by the cumulative cardiovascular toxicity of anticancer drugs (19). It has been well described that patients respond differently to the anticancer drugs and some cardiovascular toxicity observed may be fatal due to a range of predisposing factors (20). Similarly, a large registry study in Sweden showed that the patients with cancer had the highest risk of coronary heart disease in the first 6 months after their diagnosis (21) suggesting that this association is not simply by chance. The optimal time to consider CVD prevention strategies in patients with cancer is at the time of cancer diagnosis and prior to the initiation of cancer treatment. This enables oncologists to consider the risk of CVD when making cancer treatment decisions, educate patients about personalized monitoring and follow-up, reduce the burden of CVD, and improve compliance with effective cancer treatment and overall survival rate (22).

Though patients in the non-PCI group presented with different baseline characteristics compared with patients in the PCI group, on the multivariable Cox analysis, 1-year mortality rate in cancer patients with AMI was only associated with the PCI procedure which has important implications for clinical practice. Primary PCI for cancer patients with AMI has long been controversial due to the generally perceived worse prognosis of these patients than that of patients without cancer. Thus, a considerable number of patients and their families were hesitant to accept PCI in the setting of cancer. Our findings showed that whilst all-cause mortality was high, cancer patients who underwent PCI for AMI still had better short- and long-term outcomes than those who received only medical therapy. This finding is reliable because cancer patients who were not eligible for PCI due to comorbidities, fragility, or a life expectancy of less than 1 year were excluded from our study to minimize any potential bias.

Taken together, with appropriate treatment, most cancer patients can be safely carried through their AMI. With the significant improvement in cancer prognosis, especially when checkpoint inhibitor drugs that target PD-1 or PD-L1 and boost the immune response against cancer cells show a great deal of promise in treating certain cancers, attention must be paid to the optimal management of the concomitant CVD in this population. Patient-specific risk stratification and multidisciplinary team management in individuals with concomitant AMI and cancer are vital to improve the prognosis of these patients.

We acknowledge certain limitations in this study that should be highlighted when interpreting the findings. First, given the retrospective nature of the study, clinical information on certain aspect of AMI and cancer in the cohort was not available, such as the specific chemotherapy regimens of the cancer patients. Even though we excluded cancer patients who refused PCI, were deemed too sick or fragile, or who had a life expectancy less than 1 year to minimize selection bias, there still was a difference in baseline characteristics, DAPT use, and use of guideline directed medical treatment. Clinical data for Holter and ECG monitoring were not available, making it difficult to evaluate the arrhythmia burden in this population; Second, some variables were not evaluated, such as the specific drugs patients received to treat cancer. As it is well known that some anti-cancer drugs may have cardiovascular toxicity, knowledge of the patient’s cancer treatment strategy may have biased their cardiovascular treatment. Third, this is a small study conducted in a single center, therefore it is essential to develop large multi-center prospective studies for robust conclusions. Despite these limitations, this study may provide the basis for future work on the relationship between PCI and outcomes in patients with concomitant cancer and AMI.

## Conclusion

Overall, cancer patients admitted for AMI who underwent PCI had lower risk of in-hospital adverse events and mortality as well as 1-year all-cause mortality, PCI should therefore be considered for cancer patients presenting with AMI. However, chemotherapy was associated with poorer 1-year survival rate in this population.

## Data availability statement

The raw data supporting the conclusions of this article will be made available by the authors, without undue reservation.

## Ethics statement

The studies involving human participants were reviewed and approved by the Ethics Committee of The First Affiliated Hospital of Xi’an Jiaotong University (No. XJTU1AF2021LSK116) and informed consents were obtained from the patients.

## Author contributions

MT and GL: conception and design. MT, XC, YW, and HL: collection, analysis, and interpretation of data. MT, YW, and XC: drafting of the manuscript. JD and GL: revising it critically for important intellectual content. CS and GL: reviewing and editing the manuscript. All authors approved the final manuscript for submission.
